# Gene Expression Profiling Associated with Angiotensin II Type 2 Receptor-Induced Apoptosis in Human Prostate Cancer Cells

**DOI:** 10.1371/journal.pone.0092253

**Published:** 2014-03-21

**Authors:** Nana Pei, Feilong Jie, Jie Luo, Renqiang Wan, Yanling Zhang, Xinglu Chen, Zhibing Liang, Hongyan Du, Andrew Li, Baihong Chen, Yi Zhang, Colin Sumners, Jinlong Li, Weiwang Gu, Hongwei Li

**Affiliations:** 1 School of Biotechnology, Southern Medical University, Guangzhou, Guangdong, China; 2 Department of Otolaryngology-Head and Neck Surgery, Guangdong No. 2 Provincial People’s Hospital, Guangzhou, Guangdong, China; 3 Department of Neuroscience, University of Florida, Gainesville, Florida, United States of America; 4 Department of Pharmacology, University of Florida, Gainesville, Florida, United States of America; 5 Department of Physiology and Functional Genomics, University of Florida, Gainesville, Florida, United States of America; 6 Institute of Comparative Medicine and Center of Laboratory Animals, Southern Medical University, Guangzhou, Guangdong, China; Northwestern University, United States of America

## Abstract

Increased expression of angiotensin II type 2 receptor (AT2R) induces apoptosis in numerous tumor cell lines, with either Angiotensin II-dependent or Angiotensin II-independent regulation, but its molecular mechanism remains poorly understood. Here, we used PCR Array analysis to determine the gene and microRNA expression profiles in human prostate cancer cell lines transduced with AT2R recombinant adenovirus. Our results demonstrated that AT2R over expression leads to up-regulation of 6 apoptosis-related genes (TRAIL-R2, BAG3, BNIPI, HRK, Gadd45a, TP53BP2), 2 cytokine genes (IL6 and IL8) and 1 microRNA, and down-regulation of 1 apoptosis-related gene TNFSF10 and 2 cytokine genes (BMP6, BMP7) in transduced DU145 cells. HRK was identified as an up-regulated gene in AT2R-transduced PC-3 cells by real-time RT-PCR. Next, we utilized siRNAs to silence the up-regulated genes to further determine their roles on AT2R overexpression mediated apoptosis. The results showed downregulation of Gadd45a reduced the apoptotic effect by ∼30% in DU145 cells, downregulation of HRK reduced AT2R-mediated apoptosis by more than 50% in PC-3 cells, while downregulation of TRAIL-R2 enhanced AT2R-mediated apoptosis more than 4 times in DU145 cells. We also found that the effects on AT2R-mediated apoptosis caused by downregulation of Gadd45a, TRAIL-R2 and HRK were independent in activation of p38 MAPK, p44/42 MAPK and p53. Taken together, our results demonstrated that TRAIL-R2, Gadd45a and HRK may be novel target genes for further study of the mechanism of AT2R-mediated apoptosis in prostate cancer cells.

## Introduction

Prostate cancer is the most common form of cancer in North American men and is the second leading cause of cancer morbidity and mortality in the US [1], although its prognosis has improved because of advances in diagnostic and surgical techniques. To date, various kinds of therapies for patients with hormone-refractory cancer have been studied, but no effective therapy has been reported. Therefore, novel treatment strategies for prostate cancer are urgently needed.

Angiotensin II (Ang II) is the key effector in the renin-angiotensin system. Ang II has two receptors: Ang II type 1 and type 2 receptors (AT2R) [2]. AT2R, the second major receptor isoform, is primarily expressed in the mesenchyme of the fetus and to a limited extent in adult tissues [3]. It is well-established that increased expression of AT2R induces apoptosis in numerous cell lines, such as pheochromocytoma, fibroblasts, smooth muscle cells, and endothelial cells via either Ang II–dependent or Ang II–independent regulation [4]–[11]. Our previous studies revealed that AT2R over expression significantly induced apoptosis in prostate cancer cells [12]. A recent study indicates that intratracheal administration of a nanoparticle-based therapy with the AT2R gene attenuates lung cancer growth, and the effect is better than TRAIL [13]. Despite success in delineating the physiological role, the molecular and cellular actions of the AT2R-mediate apoptosis remain undefined. Here, we used real-time PCR array analysis to profile a large number of genes and microRNAs involved in AT2R induced apoptosis in prostate cancer cell lines.

In this report, amongst genes that may be related in apoptosis pathway, there are 7 apoptosis-related gene, 4 cytokines and 1 microRNA expressions that were changed in AT2R over expressed prostate cancer cells compared with control. AT2R-induced apoptosis in DU145 cells was enhanced when TRAIL-R2 was knocked down. However, the apoptotic effects mediated by AT2R were reduced in DU145 cells when Gadd45a was silenced. Interestingly, when HRK was silenced, the apoptosis induced by AT2R was reduced in PC-3 cells. Our study also indicated that the effects on AT2R-mediated apoptosis caused by down-regulation of TRAIL-R2 and HRK were independent in activation of p38 MAPK, p44/42 MAPK and p53. Our study should contribute to the identification of AT2R-sensing factors implicated in the apoptotic pathway in prostate cancer cells, and help us to understand AT2R-driven apoptosis better by providing putative target genes for further studies.

## Materials and Methods

### Cell Culture

Human prostate cancer cell lines (PC-3 and DU145 cells) were obtained from the American Type Culture Collection (Rockville, MD). PC-3 cells were cultured in F-12 medium and DU145 cells were cultured in DMEM medium supplemented with 10% FBS under 5.0% CO_2_. Sera and media were purchased from Invitrogen and American Type Culture Collection.

### Recombinant Adenoviral Construction and Preparation

Recombinant adenoviral vectors were constructed, prepared, and titrated as previously described [14]: an adenoviral vector containing the enhanced green fluorescent protein gene controlled by a cytomegalovirus promoter (Ad-CMV-EGFP) and an adenoviral vector containing genomic AT2R (G-AT2R) DNA with introns 1 and 2 and the encoding region and enhanced green fluorescent protein gene controlled by cytomegalovirus promoters (Ad-G-AT2R-EGFP).

### Cell Treatment

For viral transduction, prostate cancer cells (4×10^5^) were seeded into six-well corning tissue culture plates. On the following day, cells were transduced with Ad-G-AT2R-EGFP or the control vector Ad-CMV-EGFP and changes in cell morphology were observed using an Olympus BX41 fluorescence microscope. Transduced cells were used 24 to 48 h later, depending on the specific protocol.

For the small interfering RNA (siRNA) studies, DU145 and PC-3 cells were transfected with either TRAIL-R2, GADD45A, TP53BP2, HRK or control siRNA using Lipofectamine Reagent (Invitrogen) following the manufacturer's protocol. All siRNAs were purchased from Qiagen. These treatments were followed 24 h later by transduction with Ad-G-AT2R-EGFP (200 ifu/cell) and then, 48 h later, the number of apoptotic cells or apoptosis-related genes/proteins were examined.

### RNA Isolation and Real-Time RT-PCR

Total RNA was isolated from treated DU145 and PC3 cells using RNeasy Mini-Kit (Invitrogen) according to the manufacturer’s instructions. Isolated RNA underwent DNase I (OMEGA Biotek, Norcross, USA) treatment to remove genomic DNA. The RNA concentration was determined by an ND-1000 Spectrophotometer (Nanodrop, Rockland, DE, USA), and the RNA purity was confirmed by 260/280 optical density value of 1.8–2.0. The RNA samples were assessed for degradation status by agarose gel electrophoresis. The isolated RNA was then converted into cDNA with Oligo dT and PrimeScript Reverse Transcriptase (TAKARA, Dalian, China) regents. Fluorescent quantitative PCR was performed with the thermo-cycling condition: 95°C for 30s, followed by 40 cycles of 5s at 95°C and 34s at 60°C. The samples were analyzed with the ABI 7500 real-time PCR system (Applied Biosystems) and subjected to comparative △△C_T_ method by using human GAPDH as the internal standard. Real-time PCR products (8 µL) were loaded on 2.5% agarose gel containing ethidium bromide.

### Apoptotic Gene Expression Analysis Using Real-Time PCR Array

First-strand cDNA synthesis was performed with the RT^2^ First Strand kit (C-03) in accordance with the protocol provided by the manufacturer (SABiosciences, Frederick, MD, USA).The cDNA samples were then screened for the expression of 84 key genes involved in apoptosis by means of the Human Apoptosis RT^2^ Profiler PCR Array (PAHS-012A; Superarray, Frederick, MD, USA) on an ABI 7500 real-time PCR system (Applied Biosystems, Carlsbad, CA, USA), according to the manufacturer’s protocols. To obtain statistically data, we analyzed control and experimental samples at each of the two time intervals in triplicate. The expressions of target genes were measured relative to the mean threshold cycle (C_T_) values of five different calibrator genes (B2M, GAPDH, HPRT1, RPL13A and ACTB). The results were expressed as the relative fold change of gene expression in the AT2R conducted group compared with the control group. Genes with relative fold changes greater than ± 2 were considered as up- or down-regulated in expression. Genes that yielded a p-value of <0.05 were considered to display statistical significance for the study.

### Human Cytokines Analysis Using Real-Time PCR Array and Real-Time RT-PCR

The cDNA samples were also screened for the expression of 84 pathway-focused cytokines and by means of RT^2^ Profiler PCR Array Human Common Cytokines (PAHS-021A; Superarray, Frederick, MD, USA) on an ABI 7500 real-time PCR system (Applied Biosystems, Carlsbad, CA, USA), according to the manufacturer’s protocols. Experiments were performed once between Ad-G-AT2R-EGFP-transduced cells and Ad-CMV-EGFP-induced cells to screen cytokines generally.

Real-time RT-PCR was used to validate the expression profiling. Genes were chosen based on their possible physiological roles and on the extent to which they were regulated by the overexpression of AT2R. Oligonucleotide primers and Taqman probes specific for Bcl-2, IL6 and IL8 were obtained from Applied Biosystems. Isolation of total RNA was done as described above. Purified RNA (25 ng) was used to do RT-PCR in an Applied Biosystems Prism 7000 Sequence Detection System, with the use of One-Step RT-PCR Master Mix Reagents. Expression of the housekeeping gene GAPDH was used to normalize mRNA expression. Quantitation and analysis of gene expression was determined using the comparative cycle threshold (C_T_) method as described in Applied Biosystems User Bulletin #2. Primers used to detect the levels of other cytokines are shown in Table 1 and real-time PCR was performed using SYBR Premix Ex Taq (TAKARA) regents as described above.

**Table 1:** Primers for real-time RT-PCR.

Gene	Sense	Sequence	Product size(bp)
AT2R	Forward Reverse	5′-CCGCATTTAACTGCTCACACA-3′ 5′-ATCATGTAGTAGAGAACAGGAATTGCTT-3′	169
BMP1	Forward Reverse	5′-GCAGCAATTGGGTTGGAAAG-3′ 5′-GCGATTGAATGTGGCCATAGT-3′	90
BMP4	Forward Reverse	5′-CGGGCCAGGAAGAAGAATAAG-3′ 5′-CCAGTCATTCCAGCCCACAT-3′	78
BMP6	Forward Reverse	5′-AAGTGGTTCTCTGCCTTTTTACTATACA-3′ 5′-CTGGGCACCCTCATTTTATTTT-3′	83
BMP7	Forward Reverse	5′-CAACCTCGTGGAACATGACAA-3′ 5′-CGTGACAGCTTCCCCTTCTG-3′	100
BMP8B	Forward Reverse	5′-GGCTCAGGTCGTGAGATAGATGT-3′ 5′-ACACCACACCCTGTGATGGA-3′	99
TGFB1	Forward Reverse	5′-AGTTCAAGCAGAGTACACACAGCAT-3′ 5′-AGAGCAACACGGGTTCAGGTA-3′	84
TGFB2	Forward Reverse	5′-GACCAACCGGCGGAAGA-3′ 5′-GGGTTCGTGTATCCATTTCCA-3′	130
TGFB3	Forward Reverse	5′-ACCCCACGTGCGACAGA-3′ 5′-GGTTTGTTGCTTGTGTGTTTCC-3′	83
GADD45A	Forward Reverse	5′-GCTCTCTCCCTGGGCGACCT-3′ 5′-TCGGGGTCGCTTTCGGTCTT-3′	86
TNFRSF10B	Forward Reverse	5′-CCCAGCTGTGGAGGAGACGGT-3′ 5′-ACTACGGCTGCAACTGTGACTCCT-3′	82
TP53BP2	Forward Reverse	5′-AGTGTGGTGTGGCTCTGAACGTC-3′ 5′-GGGGGCGTTCATGACGAAGGA-3′	79
HRK	Forward Reverse	5′-CAGCCGGAGCGAGCAACAGG-3′ 5′-CCAACCGCGGCCTTTCAAGC-3′	104
GAPDH	Forward Reverse	5′-ACGGATTTGGTCGTATTGGG-3′ 5′-CGCT CCTG GAAG ATGG TGAT-3′	175

### Human MicroRNAs Analysis Using Real-Time PCR Array and Real-Time RT-PCR

To quantitate miRNA expression, total RNA was extracted from Ad-G-AT2R-EGFP-transduced and Ad-CMV-EGFP-induced cells with RNeasy Mini-Kit (Invitrogen). The isolated total RNA was reverse transcribed using the OneStep PrimeScript miRNA cDNA Synthesis Kit (Takara, Japan) according to the manufacturer’s instructions. The cDNA samples were screened for the expression 88 pathway-focused miRNAs miScript miRNA PCR Array Human Cancer PathwayFinder (MIHS-102Z; Frederick, MD, USA) on an ABI 7500 real-time PCR system.

microRNAs were analyzed further based on their possible roles in cancer and the expression that were significantly regulated in the miRNA PCR Array system. This limit was chosen to reduce the number of genes to a more workable number and to focus on those genes whose expression was changed substantially. Using these criteria, we were able to narrow our focus to 14 following miRNAs: hsa-miR-let7c; hsa-miR-let7e; hsa-miR-21; hsa-miR-125; hsa-miR-126; hsa-miR-146; hsa-miR-150; hsa-miR-182; hsa-miR-183; hsa-miR-193; hsa-miR-767; hsa-miR-149; hsa-miR-100; hsa-miR-32. Relative expression was calculated via the comparative cycle threshold (C_T_) method using the expression of U6 small nuclear RNA as the reference. The Uni-miR qPCR Primer was included in the kit. The amount of miRNA was monitored with SYBR Premix Ex Taq II (Perfect Real Time) (Takara, Japan). The PCR conditions were 30s at 95°C, followed by 40 cycles at 95°C for 5 s and 60°C for 30s.

### Apoptosis Assessment

The number of green fluorescing cells that exhibit apoptotic-like morphology induced by AT2R mediated apoptosis after transfecting siRNAs was assessed by counting cells from 10 random fields per well. Counts were done by an individual who was blinded as to the treatment.

### Western Blot Analysis

Western immunoblots were run as described previously [15]. Primary antibodies and their sources were as follows. Anti-total p38 MAPK, anti-total p53, anti-total p44/42 MAPK, anti-phosphorylated p44/42 (pp44/42) MAPK, and anti-phosphorylated p53 (pp53) were from Cell Signaling Technology. Anti-phosphorylated p38 (pp38) MAPK was from Millipore. Anti-β-actin and the secondary antibodies horseradish peroxidase-conjugated anti-rabbit IgG and anti-rabbit IgG were from Sigma-Aldrich. Anti-goat IgG was from Santa Cruz Biotechnology.

### Statistical Analysis

For all experiments, viral transduction was done in triplicate wells and repeated at least thrice. Data are presented as mean ± standard deviation (SD) from 3 to 5 independent experiments. Statistical analysis was performed with SPSS 13.0 software. All statistical analyses were performed using T-test when only 2 groups were compared, and by ANOVA when 3 or more groups were compared. P<0.05 was considered statistically significant.

## Results

### Adenoviral-Mediated Expression of AT2R in Prostate Cancer Cells

**Figure 1:**
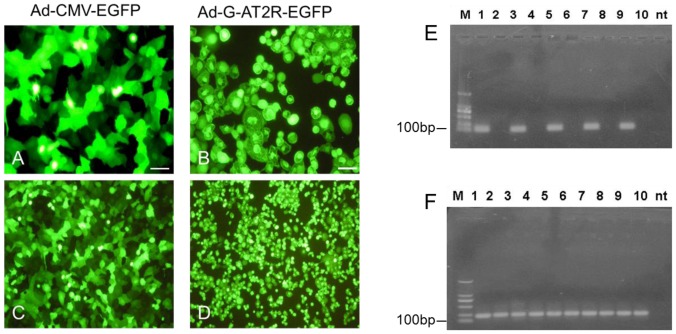
Viral vector–mediated expression of AT2R in prostate cancer cell lines. DU145 cells were transduced with Ad-G-AT2R-EGFP(B and D) and Ad-CMV-EGFP (A and C) (100 ifu/cell) for 2 days, and cell morphology was examined under a fluorescence microscope. Scale bars, 50μm. Total RNA was extracted from transduced DU145 cells and AT2Rs were detected by real time RT-PCR. Ethidium bromide–stained gels show AT2R (E) and GAPDH (F) transcripts in transduced cells. M, DL 2,000 DNA Maker (Takara); 1, 3, 5, 7, 9: transduced with 10, 20, 50, 100, 200 ifu/cell separately of Ad-G-AT2R-EGFP; 2, 4, 6, 8, 10: transduced with 10, 20, 50, 100, 200 ifu/cell separately of Ad-CMV-EGFP.

In our present study, DU145 cells infected with Ad-G-AT2R-EGFP (100 ifu/cell, 2 days) exhibited a large number of apoptotic cells compared with the control vector (Fig. 1A, B, C, D), consistent with our previous report [12]. Next, real-time PCR was used to determine the relative expression of AT2R. Our results showed that AT2R was significantly overexpressed in Ad-G-AT2R-EGFP-transduced DU145 or PC-3 cells in a dose-dependent manner (Table 2 and Fig. 1E, F).

**Table 2:** Expression of AT2Rs in transduced DU145 cells by quantitative Real-time PCR.

	△C_T_ 1	△C_T_ 2	△C_T_ 3	Mean±SD	2 ^–△C^_T_
10(ifu/cell)	–2.99	–3.05	–3.02	3.02±0.03	8.11
20(ifu/cell)	–3.02	–3.09	–2.93	3.02±0.08	8.11
50(ifu/cell)	–4.47	–4.44	–4.45	4.45±0.02	21.86
100(ifu/cell)	–6.06	–6.01	–6.02	6.03±0.03	65.34
200(ifu/cell)	–6.44	–6.65	6.61	6.57±0.03	95.01

### TRAIL-R2 and Gadd45a Contribute to AT2R-Induced Apoptosis in DU145 Cells

**Figure 2:**
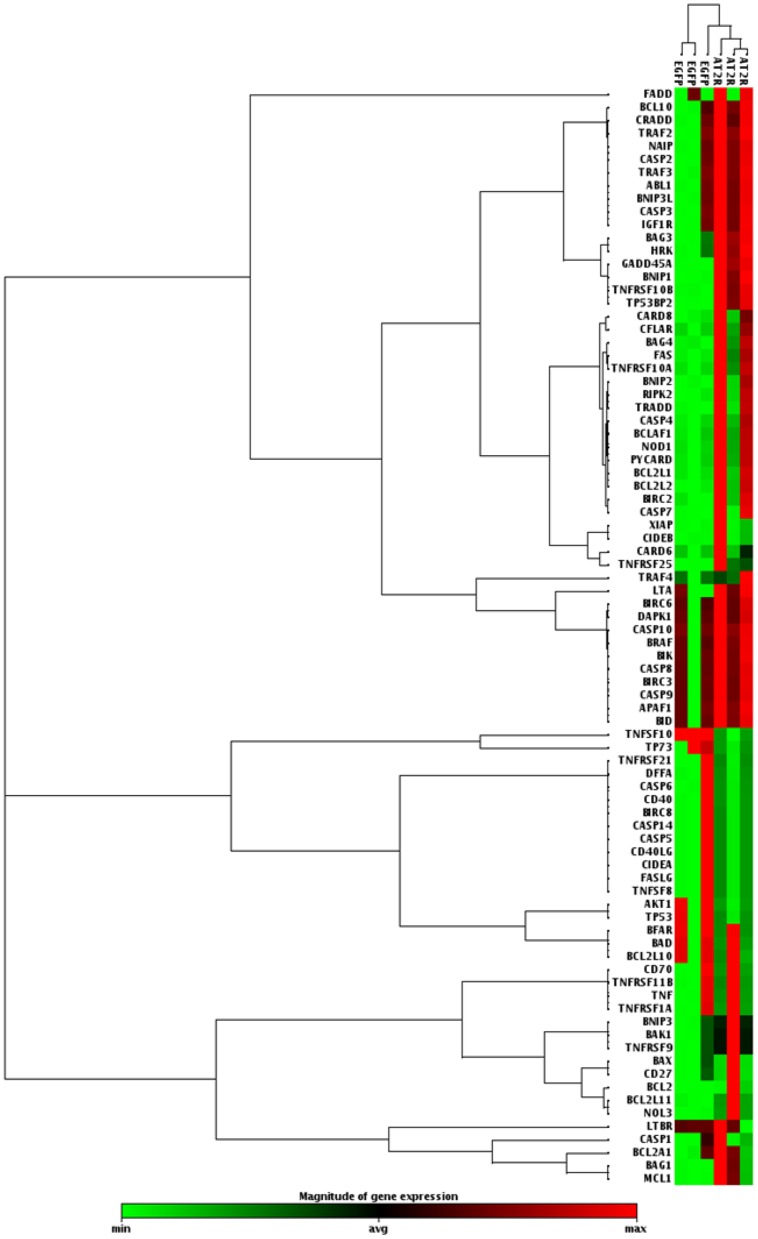
Cluster analysis of the up and down-regulated genes in DU145 cells transduced with Ad-G-AT2R-EGFP(200ifu/cell). Cells treated with Ad-CMV-EGFP (200ifu/cell) was used as a control.

**Figure 3:**
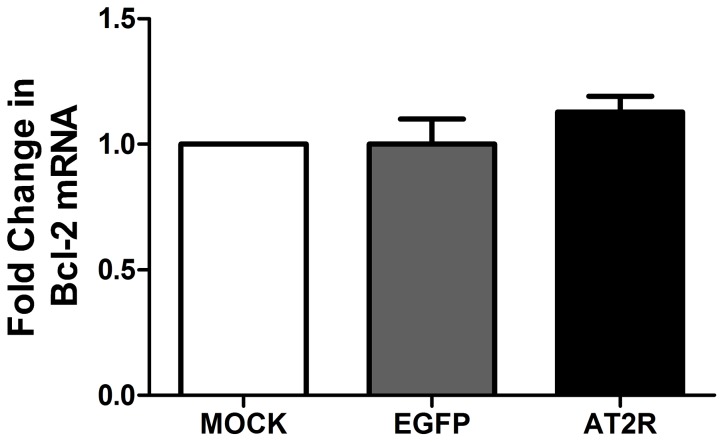
The expression of Bcl-2 in transduced DU145 cells. DU145 cells were transduced with either Ad-G-AT2R-EGFP(AT2R) or Ad-CMV-EGFP (EGFP) for 2d at 200 ifu/cell. These treatments were followed by total RNA isolation and then real-time RT-PCR analysis using specific oligonucleotide primers and Taqman probes. All data were normalized against levels of GAPDH mRNA expression within the same sample. Columns, mean from three separate experiments.

PCR Array analysis was performed to determine the molecular effects of AT2R expression in DU145 cells. Of the 84 genes represented on the Human Apoptosis RT^2^ Profiler PCR Array profiles, the expression levels of 6 genes(TRAIL-R2, BAG3, BNIPI, HRK, Gadd45a, TP53BP2 )were up-regulated and one gene(TNFSF10) was down-regulated in DU145 cells transduced with Ad-G-AT2R-EGFP (Table 3, Fig. 2). These differentially expressed genes can be allocated to genes encoding the tumor necrosis factor (TNF) ligand family (TNFSF10), the TNF receptor family (TNFRSF10B), the Bcl-2 family (BAG3, BNIP1, HRK) as well as tumor protein p53 binding protein 2(TP53BP2) and growth arrest and DNA-damage-inducible, alpha (Gadd45a). Interestingly, Bcl-2 was not regulated significantly in PCR Array analysis. Real-time PCR was further used to validity its expression. Our results showed that there was no significant change in Bcl-2 expression in Ad-G-AT2R-EGFP-transduced DU145 cells compared with Ad-CMV-EGFP-transduced cells (Fig. 3).

**Table 3:** Differentially expressed genes in human prostate cancer DU145 cells transduced Ad-G-AT2R-EGFP.

Name of gene	Description	Relative Fold Change (AT2R/control)	Accession no. (GeneBank ID)
TRAIL-R2	Tumor necrosis factor receptor superfamily, member 10b	1.95	NM_003842
BAG3	Bcl2-associated athanogene 3	3.10	NM_004281
BNIPI	Bcl2/adenovirus E1B 19kDa Interacting protein1	1.93	NM_001205
GADD45A	Growth arrest and DNA-damage-inducible,alpha	3.94	NM_001924
TP53BP2	Tumor protein p53 binding protein,2	1.96	NM_005426
HRK	Harakiri,Bcl2 interacting protein (contains only BH3domain)	3.01	NM_003806
TNFSF10	Tumor necrosis factor (ligand) superfamily, member 10	–2.05	NM_003810

**Figure 4:**
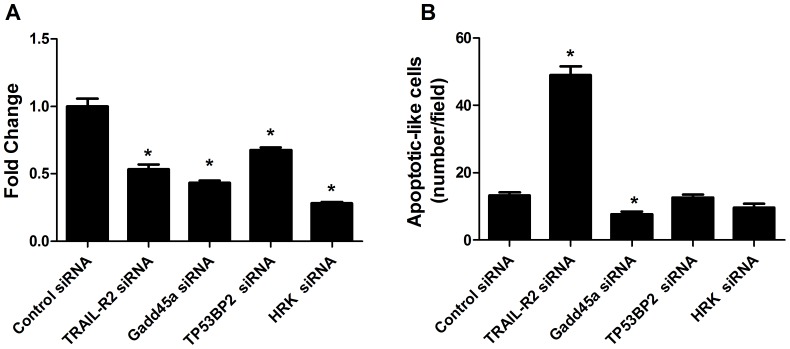
Role of apoptosis-associated genes in transduced DU145 cells. DU145 cells were transfected with TRAIL-R2,Gadd45a,TP53BP2 or HRK siRNA(20 nmol/L) or control siRNA (20 nmol/L) followed by transduction with Ad-G-AT2R-EGFP (100 ifu/cell) for 2 d. (**A**) siRNAs mediated-decrease of mRNA expression in transduced DU145 cells; (**B**) Green fluorescent cells exhibiting apoptotic morphology, which were counted from 10 fields per well.. Columns, mean of three experiments; bars, SE. *, P<0.05.

siRNA interference technology was used to further determine the role of four up-regulated genes, including TRAIL-R2, Gadd45a, TP53BP2 and HRK, in AT2R-induced apoptosis in transduced DU145 cells. Transfection of these siRNAs into DU145 cells decreased their mRNA expression (Fig. 4A). Treatment of DU145 cells with the Gadd45a siRNA reduced the apoptotic effect at about 30%, while TP53BP2 siRNA and HRK siRNA didn’t cause significant difference on AT2R-mediated apoptosis compared with controls. Interestingly, treatment of DU145 cells with the TRAIL-2 siRNA significantly increased the AT2R-induced apoptosis more than 4 times compared with control siRNA transfected cells (Fig. 4B). Moreover, the increased apoptosis was not due to the direct effect of TRAIL-R2 siRNA, since TRAIL-R2 siRNA alone caused no apoptotic results (data were not shown).

Collectively, these data indicate that the apoptosis induced by AT2R overexpression is partially dependent on Gadd45a on DU145 cells. TRAIL-R2 may be a negative regulator in AT2R-induced apoptosis in DU145 cells.

### HRK Contributes to AT2R-Induced Apoptosis in PC-3 Cells

**Figure 5:**
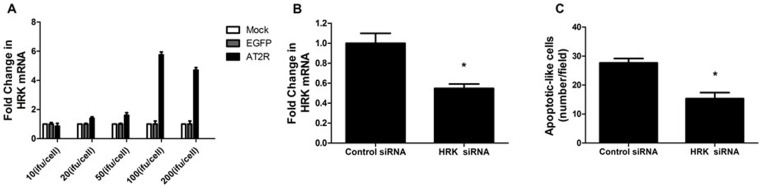
Involvement of HRK in AT2R-mediated apoptosis in PC-3 prostate cancer cells. **A,** HRK mRNA expression in PC3 cells transduced with the indicated doses. **B and C**, PC3 cells were treated with 20 nmol/L HRK siRNA or control siRNA and Ad-G-AT2R-EGFP (200 ifu/cell) as described in Materials and Methods followed 48 h later by real-time RT-PCR analysis (**B**) of either HRK mRNA or assessment of cells with apoptotic-like morphology(**C**). Columns, mean from three separate experiments in each case; bars, SE. *, P<0.05 versus control cells.

Considering the upregulated genes produced by overexpression of AT2R in DU145 cells, we next explored the effects of AT2R overexpression on the genes in PC-3 cells by real-time RT-PCR. We showed that the expression levels of HRK (a pro-apoptotic gene) were increased in PC-3 cells in a dose-dependent manner and reached a very high level at 100ifu/cell (Fig. 5A).

To explore the role of HRK in the AT2R-induced apoptosis in PC3 cells, HRK siRNA was transduced into PC-3 cells to decrease in HRK expression (Fig. 5B). The result showed downregulation of HRK in PC-3 cells significantly reduced the AT2R-induced apoptosis by ∼45% (Fig. 5C).

### The Involvement of Gadd45a, TRAIL-R2 and HRK in AT2R-Induced Apoptosis Independent of p38 MAPK, p44/42 MAPK and p53

**Figure 6:**
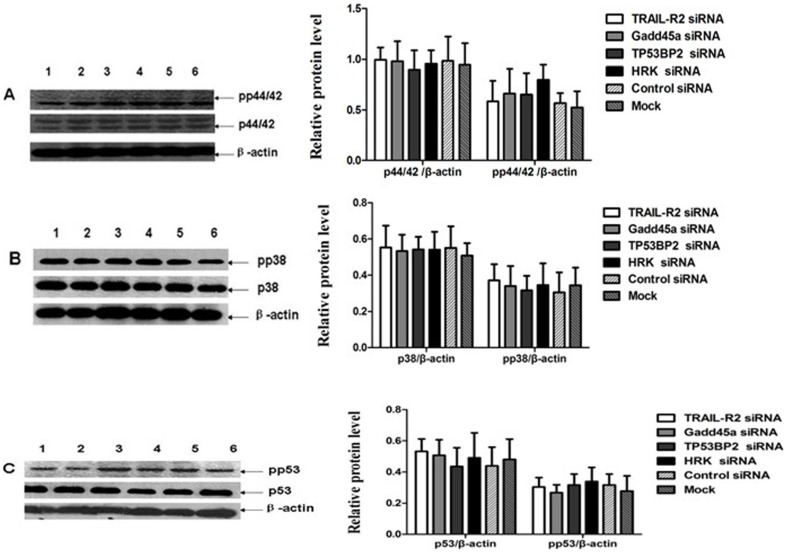
Downregulation of TRAIL-R2 and Gadd45a didn’t change the activity of p38 MAPK, p44/42 MAPK and p53 in transduced DU145 cells. DU145 cells were transfected with TRAIL-R2 siRNA *(Lane 1),* Gadd45a *siRNA (Lane 2)*, TP53BP2 siRNA *(Lane 3)*, HRK siRNA *(Lane 4)*, control *siRNA (Lane 5)* or mock *(Lane 6)* followed 24 h later by transduction with Ad-G-AT2R-EGFP (200 ifu/cell). After 2 d of incubation, cells were collected and subjected to Western blot analyses(representative of three different experiments). (A) expression levels of total p44/42, pp44/42 and β-actin protein bands. (B) expression levels of total p38, pp38 and β-actin protein bands. (C) expression levels of total p53, pp53 and β-actin protein bands.

**Figure 7:**
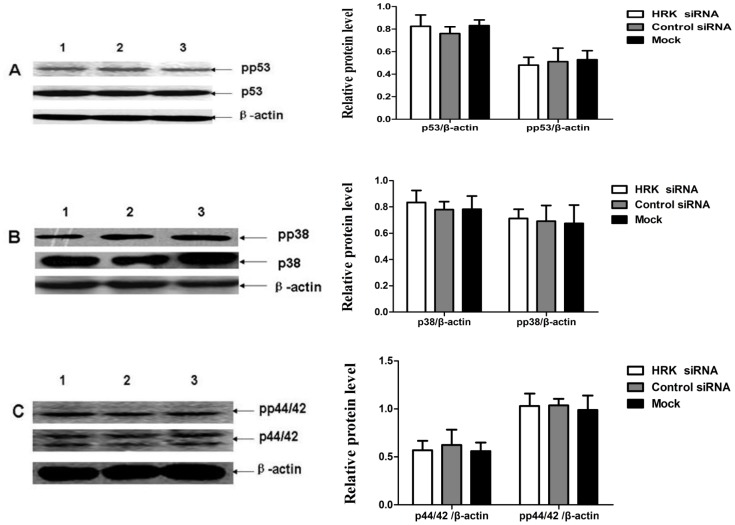
Downregulation of HRK didn’t change the activity of p38 MAPK, p44/42 MAPK and p53 in transduced PC-3 cells. PC-3 cells were transfected with HRK siRNA *(Lane1)*, control *siRNA (Lane2)* or mock *(Lane 3)* followed 24 h later by transduction with Ad-G-AT2R-EGFP (200 ifu/cell). After 2 d of incubation, cells were collected and subjected to Western blot analyses(representative of three different experiments). (**A**) expression levels of total p44/42, pp44/42 andβ-actin protein bands. (**B**) expression levels of total p38, pp38 andβ-actin protein bands. (**C**) expression levels of total p53, pp53 and β-actin protein bands.

We further investigated the downstream signaling pathway caused by AT2R over-expression on cell apoptosis. Our previous study demonstrated that increased expression of AT2R induce apoptosis through activation of the p38 MAPK pathway [14]. But in present study, the activation of p38 MAPK, p53 and p44/42MAPK were not observed in down-regulation of Gadd45a and TRAIL-R2 mediated AT2R-induced apoptosis in DU145 cells (Fig. 6A, 6B, 6C). Similar results were also observed in PC3 cells (Fig. 7A, 7B, 7C). These indicate that the involvement of Gadd45a, TRAIL-R2 and HRK in AT2R-mediated apoptosis was independent in activation of p38 MAPK, p53 and p44/42 MAPK.

### Cytokines and MicroRNAs Expression Validation

**Figure 8:**
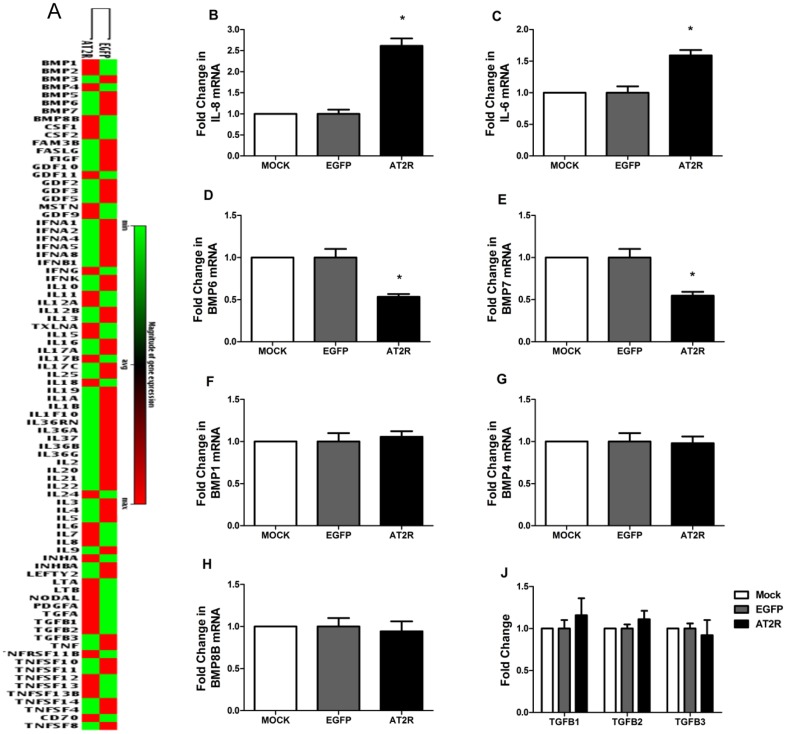
The expression of cytokines in transduced DU145 cells. DU145 cells were transduced with either Ad-G-AT2R-EGFP (AT2R) or Ad-CMV-EGFP (EGFP) for 2 d at 200 ifu/cell. Other groups of cells were mock transduced. **A**. Up and down-regulated cytokines were screened by Real-time PCR Array in DU145 cells. Real-time RT-PCR analysis of either IL8 (**B**), IL6 (**C**), BMP6 (**D**), BMP7 (**E**), BMP1 (**F**), BMP4 (**G**), BMP8B (**H**), TGFB1 (**J**), TGFB2 (**J**) and TGFB3(**J**). All data were normalized against levels of GAPDH mRNA expression within the same sample. Columns, mean from three separate experiments; bars, SE. *, P < 0.05.

**Figure 9:**
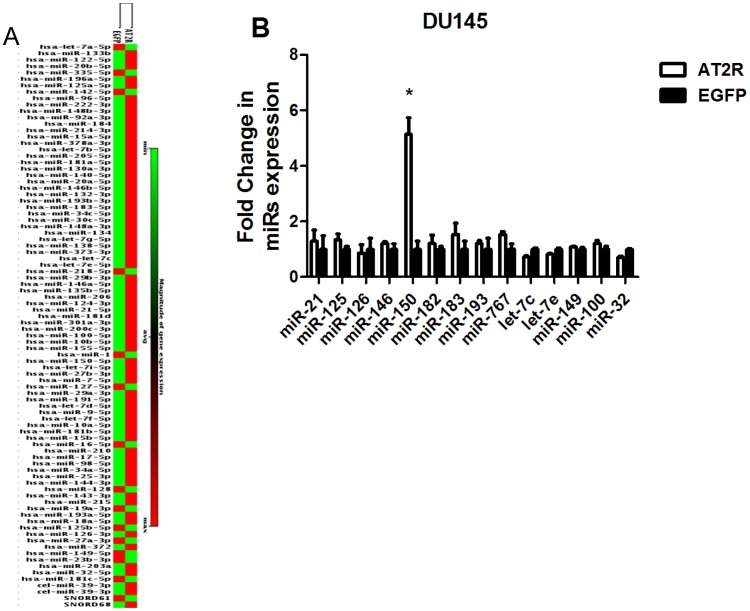
The expression of microRNAs in transduced DU145 cells. DU145 cells were transduced with either Ad-G-AT2R-EGFP (AT2R) or Ad-CMV-EGFP (EGFP) for 2 d at 200 ifu/cell. These treatments were followed by isolation of mRNA and then real-time RT-PCR analysis of selected microRNA. All data were normalized against levels U6 expression within the same sample. Columns, mean from three separate experiments; bars, SE. *, P<0.05 versus Ad-CMV-EGFP–transduced. A. Up and down-regulated microRNAs were screened by Real-time PCR Array in DU145 cells. B. Validation of the microRNA expression using Real-time PCR.

Up and down-regulated cytokines and microRNAs were screened by Real-time PCR Array in transduced DU145 cells(Fig.8A,9A). Real-time RT-PCR was used to validate the cytokine and microRNA expression profiling produced by AT2R overexpression in DU145 cells. The genes and microRNAs were chosen based on their expression changes, which were obtained from PCR array analysis, and possible physiological roles on apoptosis and proliferation. The mRNA levels of IL8 and IL6 were increased more than 2 times and 40% respectively in DU145 cells transduced with Ad-G-AT2R-EGFP compared with Ad-CMV-EGFP-transduced cells (Fig. 8B&C), while BMP6 (Fig. 8D), BMP7 (Fig. 8E) were decreased by 50% and 45% respectively and the expression of BMP1, BMP4, BMP8B, TGFB1, TGFB2 and TGFB3 (Fig.8F, 8G, 8H, 8J) were not significantly changed. With regard to microRNAs, the result indicated that microRNA 150 was increased more than 5 times in DU145 cells transduced with Ad-G-AT2R-EGFP compared with Ad-CMV-EGFP-transduced cells, while other microRNAs were not significantly changed (Fig.9B). These results were consistent with our miScript miRNA PCR Array data.

## Discussion

To the best of our knowledge, this is the first study which clearly assess apoptosis-related gene, cytokines gene and microRNA expression profiles associated with AT2R-induced apoptosis in prostate cancer cells. It is also the first study to report on the direct selective effects of Gadd45a, TRAIL-R2 and HRK on the apoptosis induced by over-expression of AT2R in DU145 or PC-3 cells. These results provide important information toward a better understanding of molecular mechanism on AT2R-mediated apoptosis in prostate cancer cells.

In this study, we observed that forced over-expression of AT2R resulted in significant changes in a large number of gene and microRNA expressions by means of PCR Array analysis. Next, we have demonstrated that TRAIL-R2 and Gadd45a are involved in apoptosis induced by AT2R in DU145 cells and HRK played an important role in AT2R–mediated apoptosis in PC-3 cells. Finally, TRAIL-R2, Gadd45a and HRK involvements in apoptosis induced by AT2R were independent in activation of p38 MAPK, p44/42 MAPK and p53 in prostate cancer cell lines.

Gadd45a was up-regulated in AT2R-overexpressed DU145 cells in our present study. Gadd45a, a p53-regulated and DNA damage-inducible gene, is a member of the Gadd45 family of genes that are known stress sensors, which modulates the cellular response to a variety of stress conditions, including genotoxic and oncogenic stress [16]-[19]. Other studies have shown that Gadd45a inhibits tumor angiogenesis via blocking of the mTOR/STAT3 pathway [20]. Gadd45a is a transcriptional target for tumor suppressor p53 and BRCA1, whose loss of function play key roles in cancer development [21]. Furthermore, Zerbini L, et al [22] have indicated that JunD, Gadd45a and Gadd45g as therapeutic targets in prostate caner. Consistent with this study, our data showed that AT2R-mediated apoptosis was partially dependent on Gadd45a in DU145 cells.

The data presented here indicate that TRAIL-R2 is involved in the AT2R-mediated apoptosis of DU145 cells. A large number of evidence showed that TRAIL, an apoptosis inducing ligand, triggers apoptosis in multiple cancer cell lines without toxicity to normal cells [23]. TRAIL has at least four cell surface receptors, and it induces apoptosis through two closely related receptors, TRAIL-R1 and TRAIL-R2 [24]. TRAIL-R1 and TRAIL-R2 induce FADD-dependent apoptosis and activate the NF-kappaB pathway[25]. Membrane-bound TRAIL/its receptors are constitutively expressed at high levels in primary and metastatic carcinomas in nearly all the patients [26]. Our study presented here showed that TNFSF10 (TRAIL) was down-regulated while TRAIL-R2 was up-regulated in AT2R-overexpressed DU145 cells. Interestingly, downregulation of TRAIL-R2 significantly enhanced the apoptotic effect induced by AT2R overexpression. Our data also showed that the apoptosis was undetectable when TRAIL-R2 was knocked down only. Hence, our experiments suggest that TRAIL and TRAIL-R2 may be negative regulators in AT2R-mediated apoptosis in DU145 cells, and the combined treatment with AT2R overexpression and TRAIL-R2 downregulation might be promising as a new gene therapy against human prostate cancer.

Some tumor cells are resistant to TRAIL-induced cytotoxicity, although TRAIL has been reported to induce apoptosis of a variety of tumor cell types [27], [28]. Failure to undergo apoptosis has been implicated in the resistance of cancer cells to TRAIL surveillance and therefore in tumor development. The molecular determinants of TRAIL-induced apoptosis have not been comprehensively examined in human prostate cancer cells. LNCaP and DU145 prostate cancer cells are resistant to TRAIL-induced apoptosis, and TRAIL was less active against them compares with PC-3 prostate cancer cells [29], [30]. The sensitivity to TRAIL-induced apoptosis could be correlated to the relative expressions of TRAIL-R1 and TRAIL-R2 versus DcR1 and DcR2 or the intracellular levels of Flame-1 [31], [32]. However, compared with LNCaP cells, which have the lowest sensitivity to TRAIL-induced apoptosis, highly sensitive PC-3 cells displayed similar or lower protein levels of TRAIL-R1 and TRAIL-R2 and higher levels of DcR2 [30]. It’s also found that the expression of TRAIL-R1 and TRAIL-R2 in the TRAIL-sensitive MCF10A cell line was not different from resistant cell lines, e.g., 184B5 [33]. This makes it unlikely that sensitivity to TRAIL-induced apoptosis is completely controlled by the relative amounts of TRAIL-R1 and TRAIL-R2. It suggests that other factors or other mechanisms may be important regulators of sensitivity to TRAIL-induced apoptosis in these cancer cells. Probably, in this study TRAIL-R2 negatively regulating AT2R-mediated apoptosis in DU145 cells will help us explore the mechanisms of sensitivity to TRAIL-induced apoptosis in different cells.

A very recent observation that TRAIL-R2, thought to only act when stimulated by TRAIL at the cell-surface, fulfils a distinct function in the nucleus where it promotes cell proliferation in a TRAIL-independent manner suggests a specific, proliferation-associated function of nuclear TRAIL-R2 [34]. Nuclear TRAIL-R2 inhibits maturation of the microRNA let-7 in pancreatic cancer cell lines and increases their proliferation. Pancreatic tumor samples have increased levels of nuclear TRAIL-R2, which correlate with poor outcome of patients [34]. These findings indicate that in the nucleus, death receptors can function as tumor promoters and might be therapeutic targets, and man help us further study the relationship between AT2R and TRAIL-R2.

Several studies have shown that HRK (pro-apoptotic BH3-only Bcl-2 family member, Harakiri) is a pro-apoptotic gene in several cells [35]–[41]. HRK inactivation is associated with a low apoptotic index in secondary glioblastomas [42]. In the present study, we showed that HRK was up-regulated in AT2R-overexpressed DU145 and PC-3 cells, and when HRK was silenced, AT2R-mediated apoptosis were significantly reduced in PC-3 but not DU145 cells. These data indicate that the apoptosis induced by AT2R over-expression is at least partially dependent on HRK in PC-3 cells. It’s also demonstrated that the expression levels of HRK were increased in PC-3 cells respectively in a dose-dependent manner. Collectively, our findings indicated that the apoptosis induced by overexpression of AT2R may be dependent on the HRK pro-apoptotic pathway in PC-3 cells. However, there are some questions to be answered such as, the mechanism of the apoptotic pathway from AT2R to HRK was not illustrated and whether or not the HRK could triggers apoptosis in other prostate cancer cells.

Our previous experiments indicate that AT2R induced apoptosis is ligand (Ang II) independent, mediated by p38 MAPK and caspase-3, and occurs via an extrinsic cell death signaling pathway [12]. However, our results showed here that p38MAPK was not involved in the role of Gadd45a, TRAIL-R2 and HRK on AT2R-induced apoptosis in DU145 and PC-3 cells. Therefore, Gadd45a, TRAIL-R2 and HRK may be located downstream of p38MAPK, or there may exist other critical pathways for AT2R-mediated apoptosis. Other studies have indicated that the apoptosis and antigrowth action induced by AT2R is mediated by down-regulation of p42/p44 MAPK, and apoptosis is considered to be an extreme result of inhibition of antigrowth signaling [43]-[47]. However, our results showed that both p44/p42 MAPK and p53 were not involved in the role of Gadd45a, TRAIL-R2 and HRK on AT2R-induced apoptosis. One unknown question that remains undiscovered is the main signaling pathways or factors in apoptosis induced by AT2R, which can be affected by Gadd45a, TRAIL-R2 and HRK in prostate cancer cells.

Bone morphogenetic proteins (BMPs) are cytokines belonging to the TGF-β superfamily, whose members reveal a variety of biologic functions such as proliferation and apoptosis [48]. Suppression of tumor formation in skin by induction of apoptosis was reported from the overexpression of BMP-6 [49]. Recent evidence has shown the tumor-suppressive effect of BMP-7 in glioma-derived cells [50]. IL-6 is a cytokine that was initially recognized as a regulator of immune and inflammatory responses, but it also regulates the growth of many tumor cells, including prostrate carcinoma [51]. Several recent reports have implicated IL-6 as an important modulator of tumor progression [52], [53]. IL-8 was originally identified as a leukocyte chemoattractant [54], but subsequent studies demonstrate that IL-8 expression enhances angiogenic activity in human bladder cancer [55]. In this study, we showed that AT2R overexpression upregulated BMP6, BMP7, IL-6 and IL-8 expression at the transcriptional level. Obviously, additional studies are needed to identify how BMP6, BMP7, IL-6 and IL-8 affect the apoptosis induced by AT2R overexpression.

MicroRNAs (miRNA) are a diverse class of small, non–protein-coding RNAs that function as critical gene regulators. Mounting evidence has shown that about half of the human miRNAs are located in cancer-associated genomic regions and function as tumor suppressor genes or oncogenes depending on their targets [56]–[58]. However, the status and function of microRNAs have never been documented in AT2R mediated apoptotic effect. Previous studies have identified aberrant expression of miR-150 in a number of malignant cells [59]–[61]. In our studies, we demonstrated that miR-150 expression was significantly increased in AT2R transduced DU145 cells. The role of miR-150 has not been previously reported in prostate cancer cells and needs to be studied further. Maybe it is an regulator in AT2R-mediated apoptosis like Gadd45a, TRAIL-R2 and HRK.

In summary, this study identified several genes and microRNAs implicated in the AT2R induced apoptotic pathway in human prostate cancer cells. These genes include not only pro-apoptotic genes but also anti-apoptotic factors, showing the highly complicated network in the AT2R-related apoptotic signaling. These results suggest that there are other pathways or factors that might be involved in the apoptotic process and should facilitate future studies on AT2R-driven apoptosis by providing putative target genes. We also believe that the combination of AT2R overexpression and TRAIL-R2 downregulation or HRK overexpresssion might be promising as a new gene therapy against human prostate cancers.
